# Neurofilament light increases over time in severe COVID-19 and is associated with delirium

**DOI:** 10.1093/braincomms/fcac195

**Published:** 2022-07-26

**Authors:** Patrick J Smeele, Lisa Vermunt, Siebe Blok, Jan Willem Duitman, AmsterdamUMC COVID-19 Biobank, Michiel van Agtmael, Anne Geke Algera, Brent Appelman, Frank van Baarle, Diane Bax, Martijn Beudel, Harm Jan Bogaard, Marije Bomers, Peter Bonta, Lieuwe Bos, Michela Botta, Justin de Brabander, Godelieve de Bree, Sanne de Bruin, David TP Buis, Marianna Bugiani, Esther Bulle, Nora Chekrouni, Osoul Chouchane, Alex Cloherty, Mirjam Dijkstra, Dave A Dongelmans, Erik Duijvelaar, Romein WG Dujardin, Paul Elbers, Lucas Fleuren, Suzanne Geerlings, Theo Geijtenbeek, Armand Girbes, Bram Goorhuis, Martin P Grobusch, Florianne Hafkamp, Laura Hagens, Jorg Hamann, Vanessa Harris, Robert Hemke, Sabine M Hermans, Leo Heunks, Markus Hollmann, Janneke Horn, Joppe W Hovius, Menno D de Jong, Rutger Koning, Endry HT Lim, Niels van Mourik, Jeaninne Nellen, Esther J Nossent, Sabine Olie, Frederique Paulus, Edgar Peters, Dan AI Pina-Fuentes, Tom van der Poll, Bennedikt Preckel, Jorinde Raasveld, Tom Reijnders, Maurits CFJ de Rotte, Job R Schippers, Michiel Schinkel, Marcus J Schultz, Femke AP Schrauwen, Alex Schuurman, Jaap Schuurmans, Kim Sigaloff, Marleen A Slim, Patrick Smeele, Marry Smit, Cornelis S Stijnis, Willemke Stilma, Charlotte Teunissen, Patrick Thoral, Anissa M Tsonas, Pieter R Tuinman, Marc van der Valk, Denise Veelo, Carolien Volleman, Heder de Vries, Lonneke A Vught, Michéle van Vugt, Dorien Wouters, A H (Koos) Zwinderman, Matthijs C Brouwer, W Joost Wiersinga, Alexander PJ Vlaar, Diederik van de Beek, Esther J Nossent, Michiel A van Agtmael, Leo M A Heunks, Janneke Horn, Harm Jan Bogaard, Charlotte E Teunissen

**Affiliations:** Neurochemistry Laboratory, Department of Clinical Chemistry, Amsterdam Neuroscience, Vrije Universiteit Amsterdam, Amsterdam UMC, Amsterdam, the Netherlands; Department of Pulmonary Medicine, Amsterdam University Medical Centre, Amsterdam 1081 HV, the Netherlands; Neurochemistry Laboratory, Department of Clinical Chemistry, Amsterdam Neuroscience, Vrije Universiteit Amsterdam, Amsterdam UMC, Amsterdam, the Netherlands; Department of Pulmonary Medicine, Amsterdam University Medical Centre, Amsterdam 1081 HV, the Netherlands; Department of Pulmonary Medicine, Amsterdam University Medical Centre, Amsterdam 1081 HV, the Netherlands; Department of Pulmonary Medicine, Amsterdam University Medical Centre, Amsterdam 1081 HV, the Netherlands; Department of Internal Medicine, Amsterdam University Medical Centre, Amsterdam 1081 HV, the Netherlands; Department of Intensive Care Medicine, Amsterdam University Medical Centre, Amsterdam 1081 HV, the Netherlands; Department of Intensive Care Medicine, Amsterdam University Medical Centre, Amsterdam 1081 HV, the Netherlands; Department of Pulmonary Medicine, Amsterdam University Medical Centre, Amsterdam 1081 HV, the Netherlands; Neurochemistry Laboratory, Department of Clinical Chemistry, Amsterdam Neuroscience, Vrije Universiteit Amsterdam, Amsterdam UMC, Amsterdam, the Netherlands

**Keywords:** neurofilament light, COVID-19, delirium, SOFA, TNF-α

## Abstract

Neurological monitoring in sedated Intensive Care Unit patients is constrained by the lack of reliable blood-based biomarkers. Neurofilament light is a cross-disease biomarker for neuronal damage with potential clinical applicability for monitoring Intensive Care Unit patients. We studied the trajectory of neurofilament light over a month in Intensive Care Unit patients diagnosed with severe COVID-19 and explored its relation to clinical outcomes and pathophysiological predictors. Data were collected over a month in 31 Intensive Care Unit patients (166 plasma samples) diagnosed with severe COVID-19 at Amsterdam University Medical Centre, and in the first week after emergency department admission in 297 patients with COVID-19 (635 plasma samples) admitted to Massachusetts General hospital. We observed that Neurofilament light increased in a non-linear fashion in the first month of Intensive Care Unit admission and increases faster in the first week of Intensive Care Unit admission when compared with mild-moderate COVID-19 cases. We observed that baseline Neurofilament light did not predict mortality when corrected for age and renal function. Peak neurofilament light levels were associated with a longer duration of delirium after extubation in Intensive Care Unit patients. Disease severity, as measured by the sequential organ failure score, was associated to higher neurofilament light values, and tumour necrosis factor alpha levels at baseline were associated with higher levels of neurofilament light at baseline and a faster increase during admission. These data illustrate the dynamics of Neurofilament light in a critical care setting and show associations to delirium, disease severity and markers for inflammation. Our study contributes to determine the clinical utility and interpretation of neurofilament light levels in Intensive Care Unit patients.

## Teaser

Smeele, Vermunt *et al*. analysed blood neurofilament light (NfL) levels in two cohorts of COVID-19 patients, showing that NfL increases over the course of Intensive Care Unit admission and may be associated with a longer duration of delirium. NfL may have potential for future neurological monitoring of Intensive Care Unit patients.

## Graphical Abstract

**Graphical Abstract:**
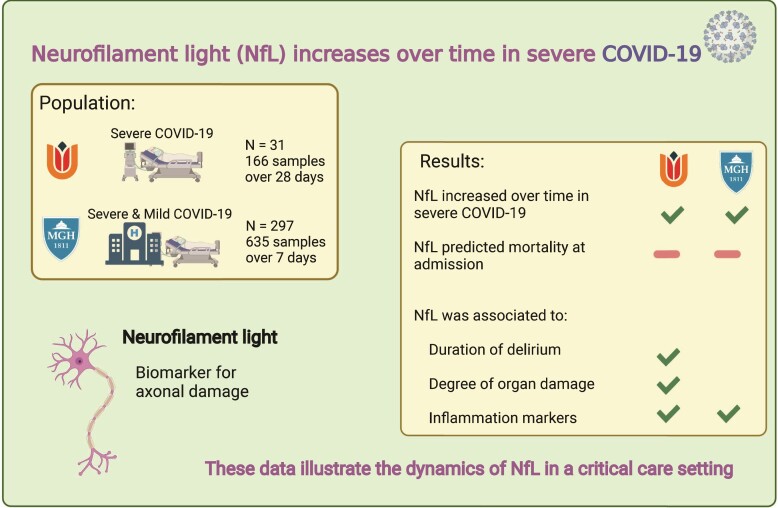

## Introduction

Neurological complications in critically ill patients prolong admission and worsen their prognosis.^1^ There is still no validated blood-based biomarker to monitor brain damage and to prognosticate neurological function in an Intensive Care Unit (ICU) setting.^2^ A novel cross-disease blood-based biomarker that is sensitive to neuronal damage is neurofilament light chain (NfL), which has been associated with disease severity and prognosis in multiple neurological diseases and general neurodegeneration.^3^ However, (neuro)biomarker studies on the ICU are often difficult due to the large heterogeneity of patients and diseases.^2^

The Coronavirus disease 2019 (COVID-19) pandemic has led to many ICU admissions sharing a similar pathology with a high incidence of neurological sequelae, such as delirium and ischaemic stroke.^4–8^ This provided an opportunity to study NfL and it’s relation to clinical and biochemical parameters using repeated sampling in a well-characterized group of patients in order to explore the dynamics of NfL and whether it could function as a prognostic marker for neurological dysfunction.

Here, we examine the trajectory of plasma NfL in ICU-treated COVID-19 patients over time, and examine its association with delirium. Secondly, we study the predictive value of NfL as a baseline marker for mortality in two independent cohorts. Lastly, we study whether markers for disease severity and inflammation at baseline are associated to the trajectory of NfL over time in these two cohorts.

## Materials and methods

### Patients

#### Amsterdam University Medical Center cohort of severe COVID-19 patients

The Amsterdam cohort was assembled as part of the Amsterdam Study for Deep Phenotyping of COVID-19 disease (Art Deco1) study. This single centre prospective cohort consisted of consecutively included, PCR-confirmed, severe COVID-19 patients at Amsterdam University Medical Centre (UMC) between March and September 2020. Patients were selected based on a minimum of 1 week of ICU treatment with mechanical ventilation due to persistent acute respiratory distress syndrome. Surplus biological samples from routine clinical practice were centrifuged at 1200 *g* and frozen at −80°C, and stored in 0.5 ml aliquots in the anonymized Amsterdam UMC COVID-19 biobank (#2020-182). Clinical data were stored using an anonymized Castor database (castoredc.com). Patients and/or their legal representatives received written information about the study and were asked to give written informed consent for participation. If direct informed consent of patients was not possible, patients could be included using a deferred consent procedure, in which case they were informed of the Biobank participation shortly after ICU discharge. Furthermore, to ensure all patients wilfully participated in the biobank, an opt-out form was sent to the patients 3 months after discharge. In case of death, informed consent was requested from the patient’s legal representatives. The study protocols were approved by the IRB of the Amsterdam UMC and biobank review committee.

#### Massachusetts General Hospital cohort of COVID-19 patients presenting to the emergency department

We performed additional data-analysis on longitudinal NfL from the publicly available Massachusetts General Hospital (MGH) COVID-19 dataset.^9^ This was a single centre prospective cohort containing patients with mild-moderate and severe COVID-19 admitted to the emergency department (ED) of MGH with PCR-confirmed COVID-19 between March and April 2020. Blood samples were collected at Day 0, Day 3 and Day 7 of hospital admission. Survival status was collected using chart review at 28 days after admission and samples were analysed for inflammation markers. An institutional IRB-approved waiver of informed consent was used. The study data were publicly released for research purposes and downloaded on 14 September 2020.^9,10^ In the MGH cohort data on mortality at 28 days was used, there was no data on delirium present.

### Nfl measurements

In the Amsterdam cohort, NfL levels were measured in both EDTA and heparin plasma using the commercially available NfL assay for SIngle MOlecule Array (SIMOA) according the kit description (NF-LIGHT™—Quanterix, Billerica, USA). For EDTA samples the result was converted by factor 1.25 in accordance to pre-analytic studies.^11^ The MGH cohort samples were analysed using the Olink® Explore 1536 platform, antibody-based multiplex technology with PCR read-out.^9^ The analytical performance of the proximity extension assay was validated for each protein assay; performance data are available at www.olink.com. Both the SIMOA and the Olink assays use the same antibodies and have been shown to be highly correlated (*r* = 0.9417 (Olink Target Neuro Exploratory—Olink).

### Clinical outcomes

Survival at 90 days and delirium during admission were recorded for the Amsterdam cohort. Incidence, duration and severity of delirium were obtained using Confusion Assessment Method-Intensive Care Unit (CAM-ICU) and Delirium Observation Score (DOS) scores from chart review. If these data were lacking, the validated Chart-Based Delirium Identification (CHART-DEL) instrument^12^ was used to supplement this data. The presence of delirium was dichotomized for the time points shortly after extubation (Day 2) and at Day 5. Duration of delirium was assessed by counting each day after extubation that a patient had a positive CAM-ICU/DOS score or registered behaviour fitting delirium criteria according to the Chart Delirium (CHART_Del) guidelines. The severity of delirium was measured 3 days after discharge to the ward using the DOS scoring system.^13^

### Biochemical markers

In the Amsterdam cohort, disease severity and organ failure were recorded using the sequential organ failure assessment (SOFA) score, of which the average score over the first 3 days of ICU admission was used. As markers of inflammation, we used plasma levels of the following key inflammatory cytokines associated with sepsis: tumour necrosis factor alpha (TNF-α), interleukin 1 beta (IL-1β), IL-6, IL-8. These were determined using a luminex platform or ELISA. For the MHG cohort no measures of SOFA scores were available. The same inflammatory blood markers (TNF-α, IL-1β, IL-6, IL-8) were selected from the available Olink data.^9^

### Clinical subgroups

In the Amsterdam cohort, all individuals had severe COVID-19 requiring at least 1 week of ICU admission. We divided them in two groups depending on 90-day survival status. Separately the MGH cohort was divided into five groups. One contained mild COVID-19 cases who did not require ventilation (*N* = 196), which was used as a control group. Two groups matched the characteristics of the Amsterdam cohort group, having severe COVID-19 requiring ICU admission of at least 7 days, of which one group survived at least 28 days (‘late discharge’, *N* = 60) and one group was deceased at 28 days (‘late mortality’, *N* = 16). In addition, there were two complementary severe COVID-19 groups in the MGH dataset: one with an ICU stay of <7 days and survival at 28 days (‘early discharge’, *N* = 7) and finally one with severe COVID-19 and mortality within 7 days (‘early mortality’, *N* = 18). Patients who deceased without ICU admission (often due to advanced directives to withhold ICU care), were not included in the analysis, leading to a total number of 297 patients.

### Statistical analysis

For the Amsterdam cohort, NfL levels were measured in pg/ml and log transformed. In the MGH cohort, NfL values were represented using Normalized Protein eXpression (NPX) units. NPX is the standard unit of the Olink PCR read-out on an inverted Log2 scale, meaning one NPX increase is a 2-fold change.

To evaluate the change of NfL over time, we used linear mixed effect models. We included a fixed effect for time after admission, a random subject slope, random subject intercept and adjusted for age and renal function (based on creatinine, measured in the same sample as the NfL measurement). Due to the non-linear trajectory of NfL measurements over time in the Amsterdam cohort, we included b-splines with minimal degrees of freedom, df = 3, essentially a cubic spline, for time in these models. With an ANOVA, we confirmed that allowing the non-linear slope improved the model fit (*P* < 0.001). We evaluated in both cohorts if the change in NfL over time differed per clinical subgroup. For this, we included a fixed effects of group and its interaction with time after admission.

For evaluation of the association between NfL with clinical outcomes, we used logistic regression models with baseline NfL and peak NfL as independent variables and mortality and delirium as dependent variables. Baseline was defined as the first sample obtained on Day 0 of admission, and peak NfL as the highest measured NfL in a given patient. Next, we determined the Spearman correlations between peak NfL and both the duration, and the severity of delirium. This was then stratified by age group, above and below 60 years^14^. All models were made with and without adjustment for age and renal function.

Lastly, we explored whether markers of inflammation and SOFA scores were associated with NfL. We explored if they might prognosticate the trajectory and peak level of NfL. Individuals were split into tertile groups based on the values of these markers. The tertile groups were then included as a fixed effect and for its interaction with time after baseline in linear mixed models for the NfL trajectory over time. In the MGH cohort, we included mild-moderate COVID-19 cases in these analyses (divided into the same tertiles) to clarify whether the relationships between NfL and mechanistic markers were specific to ICU cases. For the linear mixed models, marginal means and slope estimates generated to assess group differences in NfL levels and slopes at each time point. All models were adjusted for age and renal function. We tested the raw correlations between age, creatinine, NfL and the mechanistic markers at admission using Spearman correlation tests. The analyses were run in R version 4.0.1 using with the following statistical packages: lme4, lmerTest, emmeans.^15^

## Results

### Description of cohorts

**Figure 1:**
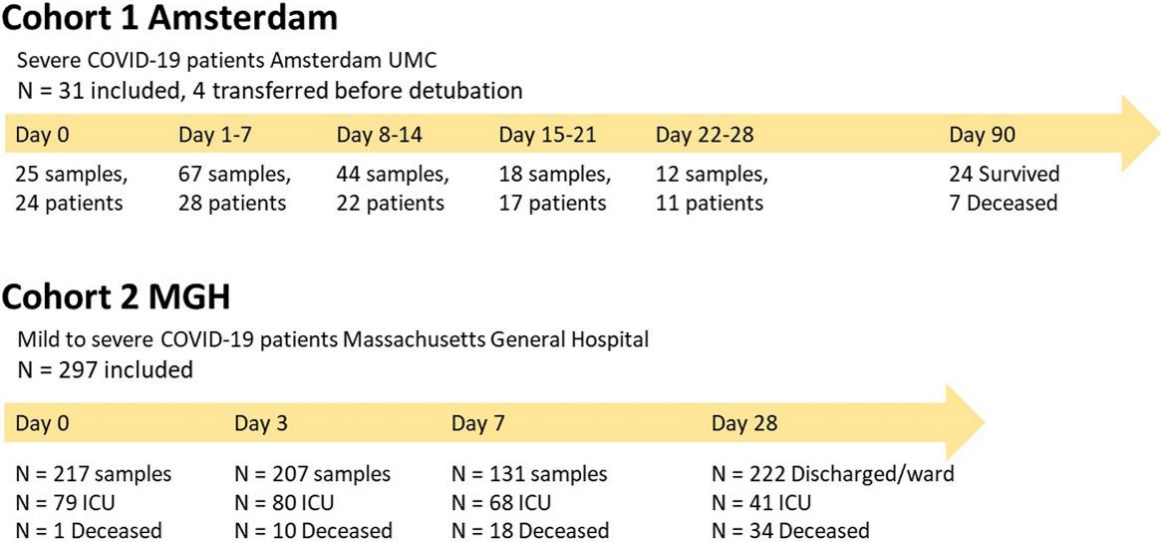
**Flow chart showing number of included patients and number of samples**. MGH, Massachusetts General Hospital.

The Amsterdam cohort consisted of 31 patients with severe COVID-19 requiring at least 1 week of ICU admission, from whom 166 blood samples were collected between Day 0 and Day 28 after admission (Fig. 1). In the Amsterdam cohort, mean age ± standard deviation was 63 ± 11 years. The majority of patients were male (74%) and mortality at 90 days was 23%.

From the MGH cohort 297 patients were included, with 635 samples obtained on Days 0, 3 and 7 after admission (Fig. 1). This included both ward patients and patients admitted to the ICU. Mortality at 28 days was 14%. For privacy reasons, data on gender were not available, and data on age were divided into categories, of which the largest age group was between 50 and 64 years (Table 1).

**Table 1:** Description of the Amsterdam cohort and the MGH cohort

	Amsterdam cohort: severe COVID-19 patients >7 days ICU	MGH cohort: all COVID-19 cases	MGH cohort: mild-moderate COVID-19 cases	MGH cohort: severe COVID-19 cases
Demographics				
Participant, *N*	31	297	217	79
Age years or *N*/category (20-34/35-49/50-64/65-79/80 + years)	63 ± 11 years	32/66/87/61/53	30/52/65/33/37	2/14/22/28/14
Male, *N* (%)	23 (74%)	—	—	—
Duration of ICU admission	28 ± 18	—	—	—
Duration of mechanical ventilation	27 ± 17	—	—	—
Days between detubation and discharge to ward	4 ± 5^a^	—	—	—
Clinical outcomes				
Mortality day 90, *N* (%)	7 (23%)	23 (8%)	NA	23 (29%)
Transferred before detubation, *N* (%)	4 (10%)	—	—	—
Presence of delirium 2 days after detubation, (Y/N)	18/4	—	—	—
Presence of delirium 5 days after detubation (Y/N)	14/7	—	—	—
Total duration delirium in days	6 ± 6.5	—	—	—
**Baseline markers (pg/mL) and SOFA score**		**Baseline markers in NPX values**		
Neurofilament light	36 ± 32	1.55 ± 1.24	1.45 ± 1.19	1.83 ± 1.32
SOFA score	8.4 ± 3.0	—	—	—
IL-1β	28.3 ± 14.1	0.64 ± 0.60	0.59 ± 0.56	0.78 ± 0.70
IL-6	296 ± 1054	5.43 ± 1.70	4.95 ± 1.42	6.72 ± 1.73
IL-8	89 ± 105	6.60 ± 1.12	6.37 ± 1.00	7.21 ± 1.22
TNF-α	14 ± 6.5	1.87 ± 0.60	1.81 ± 0.53	2.02 ± 0.74

Mean ± SD unless otherwise specified.

Pg/ml, pictogram per mililitre; NPX, Normalized Protein eXpression, Log2 scale; ICU, Intensive Care Unit; SOFA, Sequential Organ Failure Assessment; IL, interleukin; TNF, tumour necrosis factor.

^a^ Excluding one outlier with 66 days between detubation and discharge to ward.

### Nfl trajectory increased over time on the ICU

**Figure 2:**
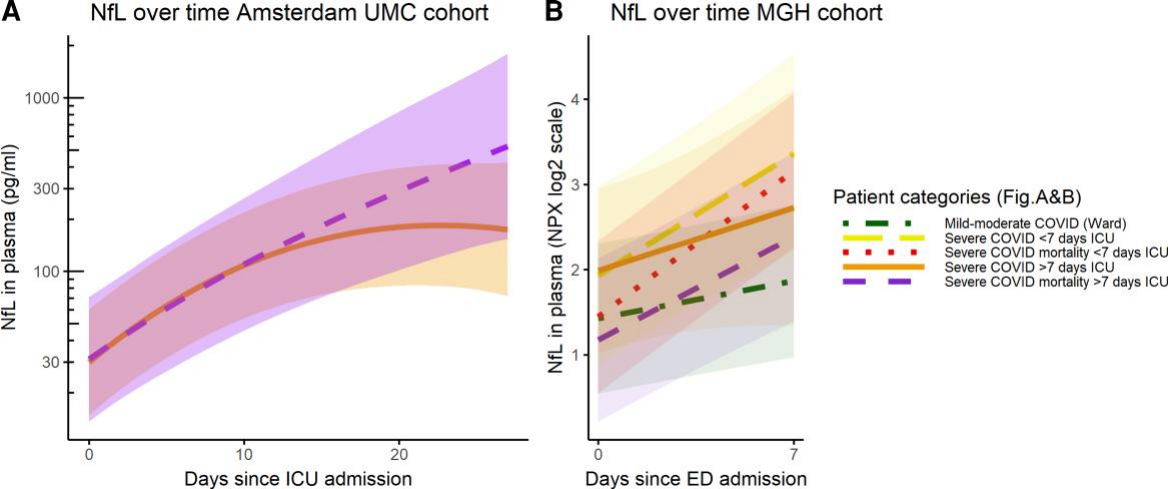
**NfL over time in COVID-19**. (**A**) ICU patients with >7 days of admission, admitted to Amsterdam UMC. Orange/solid line: survival at 90 days, purple/dashed line: deceased at 90 days. Linear mixed model, marginal means show slopes of survival group stopped increasing on Day 19 and the slope of the mortality group stopped increasing on Day 25, these slopes differed between Days 17 and 24 (*t*-ratio = −2.165, *P* = 0.03 and *t*-ratio = −2.06, *P* = 0.04, respectively) (**B**) Patients admitted to the ED of MGH. Analysed using linear mixed models: green/dot-dashed line: mild-moderate COVID-19 (used as reference, slope *t*-value = 4.422, *P* < 0.001), yellow/longdash: severe COVID-19 with ICU admission and discharge before Day 7 (slope *t* = 2.53 *P* = 0.01). Red/dotted line: severe COVID-19 with ICU admission and mortality before Day 7 (slope *t* = 0.54, *P* = 0.59). Orange/solid line: severe COVID-19 with ICU admission and discharge after Day 7 (slope *t* = 8.32, *P* < 0.001). Purple/dashed line: severe COVID-19 with ICU admission and mortality after Day 7 (slope *t* = 3.23, *P* = 0.001).

NfL increased over time in both cohorts (Amsterdam *P* < 0.0001; MGH *P* < 0.0001). In the Amsterdam cohort, NfL levels stopped increasing significantly at Day 22 (*P* < 0.05); indicating a plateau. Group level NfL values [marginal mean (95% confidence interval)] increased from 30 (23–39) pg/ml at ICU admission to 78 (60–102) pg/ml on Day 7, 147 (107–201) pg/ml on Day 14 and 208 (138–313) pg/ml on Day 21. The NfL trajectory differed depending on the clinical outcome. In survivors NfL stopped increasing significantly at Day 19 [NfL 177 (111–283) pg/ mL] and in non-survivors on Day 25 [NfL 446 (160–1244 pg/ml)] (Fig. 2, Supplementary Fig. 2).

Similarly, to the Amsterdam cohort, NfL increased between Day 0 and Day 7 in all severe COVID-19 patients in the MGH cohort. The exception to this was the early mortality group (before Day 7), which had the highest NfL at admission, and did not increase over time (*β* ± SE, 0.8120 ± 0.280, *P* = 0.0323, Fig. 2 and Supplementary Fig. 2). In the MGH cohort, the late mortality group (after Day 7) had the lowest NfL at admission, significantly lower than the early mortality group. The other groups (mild-moderate and discharged from ICU) did not significantly differ from each other at baseline. NfL increased faster in the groups with a long ICU stay; the late mortality (*P* < 0.0001) and late discharge group (*P* = 0.0131) when compared with the mild-moderate group. In the two groups with a short stay; those with mortality before Day 7 or discharge before Day 7, the trajectory of NfL did not deviate significantly from the mild-moderate group (Fig. 2 and Supplementary Fig. 2).

### Nfl is related to delirium, but not mortality

**Figure 3:**
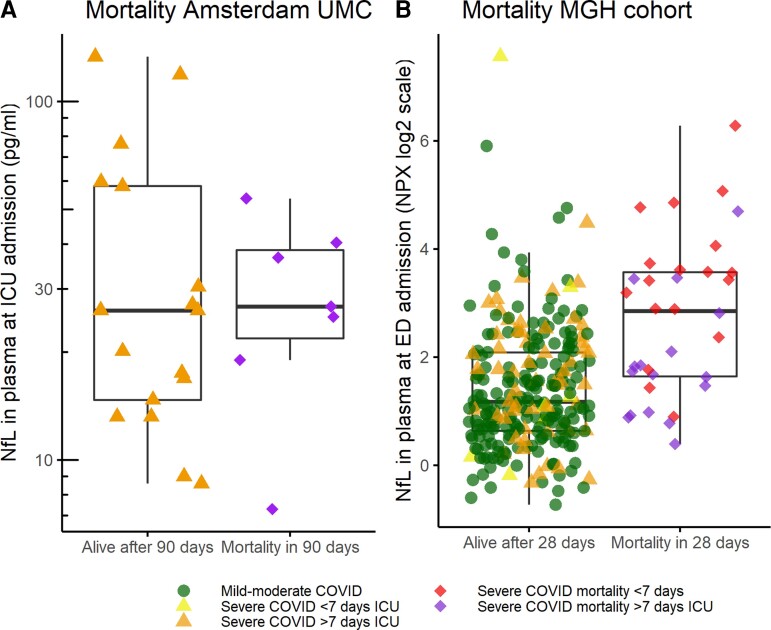
**Baseline NfL in relation to mortality**. (**A**) Mortality at 90 days in Amsterdam cohort. Orange triangle = survival after >7 days ICU, purple diamond = mortality after >7 days ICU (analysed using logistic regression, *Z* = −0.34, *P* = 0.72). (**B**) Mortality at 28 days in MGH cohort (logistic regression, *Z* = −0.96, *P* = 0.34). Green circle: Mild-moderate COVID-19, yellow triangle: survival after <7 days ICU, orange triangle: survival after >7 days ICU, purple diamond: mortality after >7 days ICU, red diamond: mortality after <7 days ICU.

In the Amsterdam cohort, NfL levels at admission and peak NfL levels did not predict mortality at 90 days (*P* = 0.729 and *P* = 0.613 respectively; Fig. 3A) this did not change when correcting for age and renal function. In the MGH cohort NfL at ED presentation predicted mortality with an odds ratio (OR) of 2.08 ± 1.16, *P* ≤ 0.001. However, when corrected for age and renal function, the odd ratio decreased and was no longer significant (OR 1.08 ± 1.29 *P* = 0.763; Fig. 3B). NfL levels on Day 7 did not predict mortality at 28 days in the MGH cohort.

**Figure 4:**
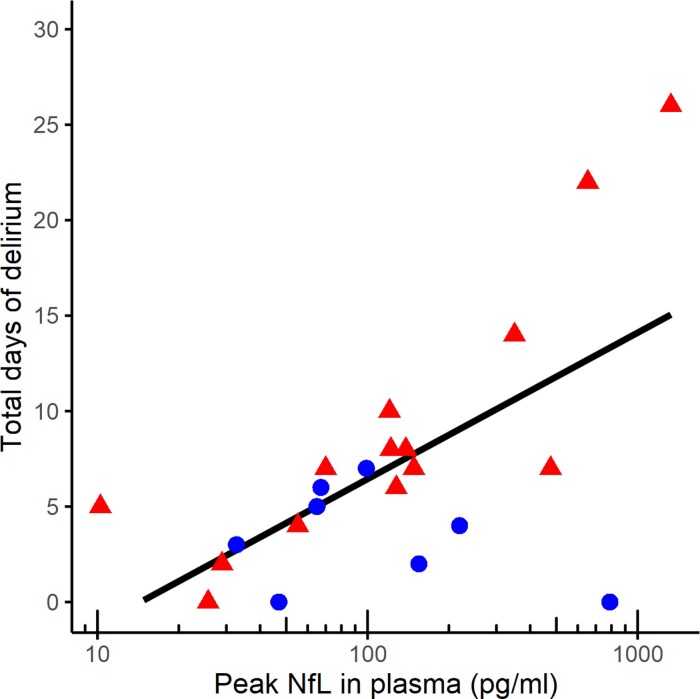
**Peak NfL levels in relation to length of delirium in days in the Amsterdam cohort**. Spearman correlation, *ρ* = 0.5, *P* = 0.02. Patients above 60 years of age in red triangle, and below 60 years of age in blue circle.

In the Amsterdam cohort, peak NfL levels did not predict which patients developed delirium directly after extubation (*P* = 0.7348), nor 5 days later (*P* = 0.184). However, a correlation was seen between peak NfL levels and total duration of delirium (*ρ* = 0.5, *P* = 0.0172). When stratified according to age (<60 years versus ≥60 years), this effect was driven by those older than 60 years (*ρ* = 0.8, *P* ≤ 0.001; Fig. 4). Peak NfL levels were not associated to DOS scores on the ward (*ρ* = 0.4, *P* = 0.0724), this did not change when stratified according to age (*ρ* = 0.53 *ρ* = 0.0609 in those older than >60 years).

### Nfl increases were related to the SOFA score and inflammation

**Figure 5:**
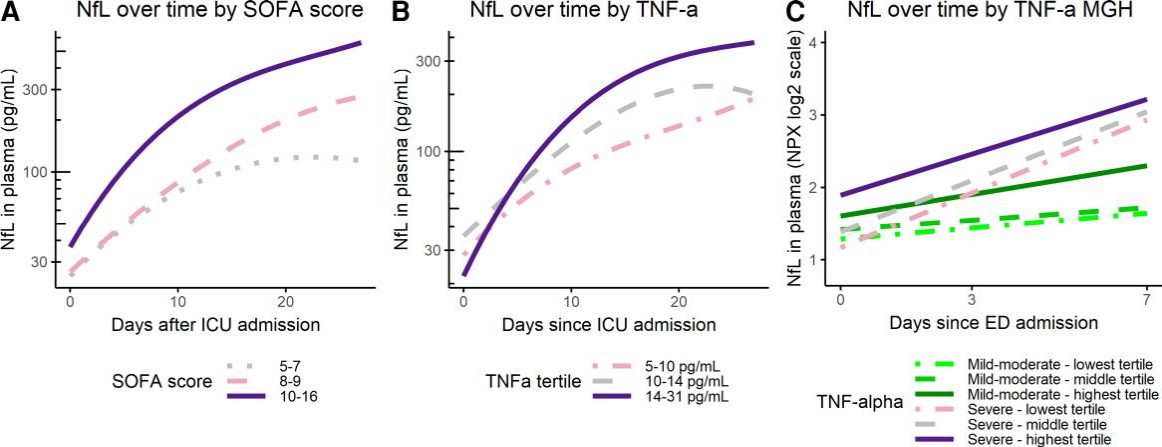
**NfL in relation to other factors**. Analysed using linear mixed models, the lowest tertile group of the factor of interest acting as reference group. Marginal means to test the days when slopes differed significantly between the tertile groups (**A**) SOFA score in the AmsterdamUMC cohort (average score over the first 3 days of admission used). Slopes differ significantly between lowest and highest tertile from Day 8 onwards (*t*-ratio = −2.55, *P* = 0.04). (**B**) TNF-α in the AmsterdamUMC cohort. Slopes differ significantly between the highest and middle tertile from Day 0 until Day 6 (*t*-ratio = −2.38, *P* = 0.05, *t*-ratio = −2.42, *P* = 0.05, respectively), between the highest and lowest tertile between Day 2 and Day 8 (*t*-ratio = −2.64, *P* = 0.02 and *t*-ratio = −2.66, *P* = 0.03, respectively). (**C**) TNF-α in the MGH cohort. Linear mixed model: mild-moderate COVID-19 group lowest tertile used as reference group (*t*-value = 2.09, *P* = 0.04). Severe COVID-19 lower tertile (*t*-value = 5.77), middle tertile (*t*-value = 5.15), upper tertile (*t*-value = 4.23), all *P*-values < 0.001. Using marginal means, the differences in slopes per group were analysed. The severe COVID-19 tertile groups had significantly steeper slopes than the mild-moderate COVID-19 tertiles groups (*t*-ratio between −6.30 and −2.91, all *P*-values < 0.01), except the slope of the highest mild-moderate COVID-19 tertile did not differ from the highest severe COVID-19 tertile (*t*-ratio −2.57, *P* = 0.108).

Patients in the highest tertile of SOFA scores (Score: 10–16) showed a steeper rise of NfL over time in the first 5 days, when compared with the lowest SOFA tertile (Score: 5–7). NfL stopped increasing in the highest SOFA tertile as early as Day 13 and decreased on Days 18–19. NfL values of the middle SOFA tertile fell between both groups, but did not differ significantly from either of them (Fig. 5A).

Among the inflammation markers TNF-α, IL-1β, IL-6 and IL-8, only baseline TNF-α and IL-1β values showed associations with the NfL trajectory in the Amsterdam cohort. Specifically, the highest TNF-α tertile exhibited a faster increase in NfL levels compared with the middle (Days 0–5) and lower TNF-α tertile (Days 2–8, Fig. 5B). NfL also increased faster in the highest IL-1β tertile as compared with the lowest IL-1β tertile (Days 6–9; Supplementary Fig. 3A). For the MGH cohort, comparable tertile groups were created according to TNF-α, IL-1β, IL-6 and IL-8 at admission for both mild and severe COVID-19. When comparing the inflammation markers of the severe COVID-19 and mild COVID-19 subgroups of the MGH cohort, the severe COVID-19 groups showed a consistent pattern of faster increasing NfL (Supplementary Fig. 3E–H). This effect was present regardless of subdivision according to IL-1β, IL-6 and IL-8 tertiles. The exception to this was TNF-α, where the highest tertile of mild COVID-19 patients had an increase in NfL similar to the severe COVID-19 groups (Fig. 5C). Baseline NfL levels were mostly comparable between the TNF-α tertiles, only the highest severe COVID-19 TNF-α tertile group was elevated compared with the lower tertile of the severe COVID-19 group, and the middle and lower mild COVID-19 group (Fig. 5C).

Spearman correlations showed that in the Amsterdam cohort creatinine and IL-8 were correlated at baseline (*ρ* = 0.54) and SOFA scores were correlated to IL-8 and duration of ventilation (*ρ* = 0.54 and 0.49, respectively). In the MGH cohort most markers show a weak or weak to moderate positive correlation. NfL correlated to age (*ρ* = 0.65), creatinine (*ρ* = 0.44) and TNF-α (*ρ* = 0.46). Furthermore IL-6 and IL-8 were correlated (*ρ* = 0.48) at baseline (*ρ* = 0.65; Supplementary Fig. 1).

## Discussion

In this study, we examined NfL during the course of admission in critically ill COVID-19 patients. Baseline NfL levels were comparable between ward and ICU patients and were not predictive of mortality. However, NFL increased steeply in ICU patients and peak levels were associated to a longer duration of delirium. This implies that the trajectory of NfL and it’s cumulative value could be valuable for signalling neurological distress in an ICU setting. Lastly, we identified several factors that associated with subsequent NfL increases, which could guide further explorations into the underlying pathophysiological mechanisms of increased NfL in COVID-19 patients on the ICU.

Our observation that NfL in blood increased during a severe COVID-19 infection is supported by previous studies.^16,17^ Others have shown that within ICU patients, COVID-19 is associated with higher NfL when compared with non-COVID-19 ICU controls, after correcting for age and comorbidities.^18,19^ A previous longitudinal analysis of NfL in COVID-19 patients showed temporal changes with no clear increasing trend for the 35 patients (both ICU and ward) with the highest measured NfL in their cohort.^20^ This contrast with our findings could be explained due to our cohort containing only ICU patients with a long duration of admission. However, these discrepancies warrant future investigation.

In line with previous work in other settings, we found a relationship between increased NfL and delirium, which suggests that NfL levels captured neurological distress.^21–24^ The dynamics of NfL observed in our study indicate that NfL might have utility as a tool to monitor neurological damage in the future for sedated patients. Repeated NfL measurements could also have an application as a surrogate biomarker to monitor the effect of interventions that prevent neurological complications on the ICU.^25^ Lastly, peak NfL could have utility in prognosticating both short term and long-term outcomes, as we found a relation with delirium after extubation and others with the length of rehabilitation after discharge.^21–24,26–28^

Contrary to previous studies, baseline NfL was not predictive for mortality in our study.^29,30^ This could result from the fact that the Amsterdam cohort was small and only included patients with at least a week admission to the ICU. Previously an association between NfL and mortality was seen in ICU patients, in this case samples had been drawn 48 h after admission.^18^ The MGH cohort was larger, and also did not show a relation with survival after correction for age and renal function. Still, within the MGH cohort those with mortality before Day 7 did have the highest NfL levels at baseline, which may be an indication that NfL at admission might be a predictor of fast mortality, rather than for the complete disease course.

There are several reasons why NfL could be increased in the blood stream in COVID-19 patients. Previous studies identified toxic/metabolic encephalopathy as the most common neurologic complication in COVID-19.^31^ The observed relation between NfL and SOFA scores in our study and by others could argue in favour of this cause of NfL release.^19^ Another potential mechanism is that NfL increases are caused by axonal damage due to the inflammatory response of the brain to COVID-19. TNF-α is an inflammatory marker, that in particular has been shown to be an independent predictor of disease severity and death in COVID-19.^32^ In our study, TNF-α levels were the most consistent predictor of NfL increases.

Clinical implementation of NfL on the ICU would warrant establishing cut-off values for abnormality and the earliest detectable increase. This might be complicated by the complex kinetics of biological markers in critical care patients, e.g. sedatives, other medication, comorbidities and possibly the type of brain insult.^19,33^ Previous studies found NfL levels to increase by over 100 pg/ml after an acute event such as stroke or cardiac arrest, indicating that an effect can be detected within 24 h of onset after acute brain damage.^27,28,34,35^ In the case of COVID-19 sepsis elevations appear to be less pronounced and at a slower rate. The potential diagnostic value of a monitoring approach with NfL might be established by comparison to the current standards MRI and EEG to detect severe complications. Finally, peripheral neuropathy is common on the ICU and has been shown to increase neurofilament proteins.^36^ Further studies using multiple neurobiomarkers, such as GFAP, UCH-L1, NSE in combination with functional scores and possibly EMG data could aid in unravelling the specificity of changes and when, and to what extent, NfL increases are of peripheral or central origin. Lastly renal clearance is an important factor to consider in future analysis.

The study had several strengths and limitations. The Amsterdam cohort was relatively small and lacked a non-COVID-19 and non-ICU control group. However, the homogeneity and long period of repeated sampling of NfL allowed for more power to evaluate the biomarker dynamics.^29^ As renal clearance is an important factor, it is a limitation that there were no dialysis patients in the Amsterdam cohort. The role of treatment effects was not explored in this paper. A previous study exploring this noted that Remdesivir may influence NfL levels.^20^ Both cohorts were collected during the first wave of the pandemic when Remdesivir was not approved for use. However, the effect of COVID-19 treatments on NfL remain an interesting topic for future research. The MGH cohort had non-ICU and short-stay ICU controls, the limitation with this cohort, however, was that the sampling period was 7 days. Secondly, the lack of non-COVID-19 ICU controls makes it difficult to determine to what extent the effects were specific to COVID-19. Further limitations related to the accuracy of how delirium screening tools were used during the pandemic both on the ICU and the ward. In case of lacking data we applied the Chart_Del system for chart review, which is an approach supported by previous literature.^37^

In conclusion, the availability of a biomarker for neurological monitoring of ICU patients would be of significant value in clinical decision making. Our results show that NfL increases during admission with severe COVID-19 and relates to delirium, supporting the notion that NfL levels in plasma may have utility in a critical care setting.

## Supplementary Material

fcac195_Supplementary_Data

## Data Availability

All data available upon request. Email: E-mail: c.teunissen@amsterdamumc.nl. The syntax of the analysis is provided in the Supplementary Materials (page 16). The Olink data from MGH can be found at https://www.olink.com/mgh-covid-study.

## Appendix

*Amsterdam UMC COVID-19 Biobank investigators:* Michiel van Agtmael^2^, Anne Geke Algera^1^, Brent Appelman^2^, Frank van Baarle^1^, Diane Bax^3^, Martijn Beudel^4^, Harm Jan Bogaard^5^, Marije Bomers^2^, Peter Bonta^5^, Lieuwe Bos^1^, Michela Botta^1^, Justin de Brabander^2^, Godelieve de Bree^2^, Sanne de Bruin^1^, David T.P. Buis^1^, Marianna Bugiani^5^, Esther Bulle^1^, Nora Chekrouni^4^, Osoul Chouchane^2^, Alex Cloherty^3^, Mirjam Dijkstra^12^, Dave A. Dongelmans^1^, Erik Duijvelaar^5^, Romein W.G. Dujardin^1^, Paul Elbers^1^, Lucas Fleuren^1^, Suzanne Geerlings^2^, Theo Geijtenbeek^3^, Armand Girbes^1^, Bram Goorhuis^2^, Martin P. Grobusch^2^, Florianne Hafkamp^3^, Laura Hagens^1^, Jorg Hamann^7^, Vanessa Harris^2^, Robert Hemke^8^, Sabine M. Hermans^2^, Leo Heunks^1^, Markus Hollmann^6^, Janneke Horn^1^, Joppe W. Hovius^2^, Menno D. de Jong^9^, Rutger Koning^4^, Endry H.T. Lim^1^, Niels van Mourik^1^, Jeaninne Nellen^2^, Esther J. Nossent^5^, Sabine Olie^4^, Frederique Paulus^1^, Edgar Peters^2^, Dan A.I. Pina-Fuentes^4^, Tom van der Poll^2^, Bennedikt Preckel^6^, Jorinde Raasveld^1^, Tom Reijnders^2^, Maurits C.F.J. de Rotte^12^, Job R. Schippers^5^, Michiel Schinkel^2^, Marcus J. Schultz^1^, Femke A.P. Schrauwen^12^, Alex Schuurman^10^, Jaap Schuurmans^1^, Kim Sigaloff^1^, Marleen A. Slim^1,2^, Patrick Smeele^5^, Marry Smit^1^, Cornelis S. Stijnis^2^, Willemke Stilma^1^, Charlotte Teunissen^11^, Patrick Thoral^1^, Anissa M Tsonas^1^, Pieter R. Tuinman^1^, Marc van der Valk^2^, Denise Veelo^6^, Carolien Volleman^1^, Heder de Vries^1^, Lonneke A. Vught^1,2^, Michéle van Vugt^2^, Dorien Wouters^12^, A. H (Koos) Zwinderman^13^, Matthijs C. Brouwer^4^, W. Joost Wiersinga^2^, Alexander P.J. Vlaar^1^, Diederik van de Beek (d.vandebeek@amsterdamumc.nl)^4^.

See Supplementary Materials for full list including affiliations.

## Abbreviations

CAM-ICU =: Confusion Assessment Method-Intensive Care Unit
CHART_DEL =: Chart Delirium
COVID-19 =: COrona VIrus Disease 2019
DOS =: Delirium Observation Score
ED =: Emergency Department
ICU =: Intensive Care Unit
IL =: Interleukin
MGH =: Massachusetts General Hospital
NfL =: Neurofilament Light
SIMOA =: SIngle MOlecule Array
SOFA =: Sequential Organ Failure Assessment
TNF-α =: Tumour Necrosis Factor alpha
UMC =: University Medical Centre
