# Supplementary material for: Neurofilament light increases over time in severe COVID-19 and is associated with delirium
Source: Brain Commun. 2022 Jul 26;4(4):fcac195. doi: 10.1093/braincomms/fcac195 (PMC9351727; doi:10.1093/braincomms/fcac195)
Supplement: fcac195_Supplementary_Data [file fcac195_supplementary_data.zip › Supplementary_material AmsterdamUMC COVID-19 Biobank Investigators.pdf]

AmsterdamUMC COVID-19 Biobank Investigators – including author affiliations

Michiel van Agtmael[2], Anne Geke Algera[1], Brent Appelman[2], Frank van Baarle[1], Diane Bax[3], Martijn Beudel[4], Harm Jan Bogaard[5], Marije Bomers[2] Peter Bonta[5], Lieuwe Bos[1], Michela Botta[1], Justin de Brabander[2], Godelieve de Bree[2], Sanne de Bruin[1], David T.P. Buis[1], Marianna Bugiani[5], Esther Bulle[1], Nora Chekrouni[4], Osoul Chouchane[2] Alex Cloherty[3], Mirjam Dijkstra[12], Dave A. Dongelmans[1], Erik Duijvelaar[5], Romein W.G. Dujardin[1], Paul Elbers[1], Lucas Fleuren[1], Suzanne Geerlings[2] Theo Geijtenbeek[3], Armand Girbes[1], Bram Goorhuis[2], Martin P. Grobusch[2], Florianne Hafkamp[3], Laura Hagens[1], Jorg Hamann[7], Vanessa Harris[2], Robert Hemke[8], Sabine M. Hermans[2] Leo Heunks[1], Markus Hollmann[6], Janneke Horn[1], Joppe W. Hovius[2], Menno D. de Jong[9], Rutger Koning[4], Endry H.T. Lim[1], Niels van Mourik[1], Jeaninne Nellen[2], Esther J. Nossent[5], Sabine Olie[4], Frederique Paulus[1], Edgar Peters[2], Dan A.I. Pina-Fuentes[4], Tom van der Poll[2], Bennedikt Preckel[6], Jorinde Raasveld[1], Tom Reijnders[2], Maurits C.F.J. de Rotte[12], Job R. Schippers[5], Michiel Schinkel[2], Marcus J. Schultz[1], Femke A.P. Schrauwen[12], Alex Schuurman[10], Jaap Schuurmans[1], Kim Sigaloff[1], Marleen A. Slim[1,2], Patrick Smeele[5], Marry Smit[1], Cornelis S. Stijnis[2], Willemke Stilma[1], Charlotte Teunissen[11], Patrick Thoral[1], Anissa M Tsonas[1], Pieter R. Tuinman[1], Marc van der Valk[2], Denise Veelo[6], Carolien Volleman[1], Heder de Vries[1], Lonneke A. Vught[1,2], Michéle van Vugt[2], Dorien Wouters[12], A. H (Koos) Zwinderman[13], Matthijs C. Brouwer[4], W. Joost Wiersinga[2], Alexander P.J. Vlaar[1], Diederik van de Beek (d.vandebeek@amsterdamumc.nl)[4].

1Department of Intensive Care, Amsterdam UMC, Amsterdam, The Netherlands; 2Department of Infectious Diseases, Amsterdam UMC, Amsterdam, The Netherlands; 3Experimental Immunology, Amsterdam UMC, Amsterdam, The Netherlands; 4Department of Neurology, Amsterdam UMC, Amsterdam Neuroscience, Amsterdam, The Netherlands; 5Department of Pulmonology, Amsterdam UMC, Amsterdam, The Netherlands; 6Department of Anesthesiology, Amsterdam UMC, Amsterdam, The Netherlands; 7Amsterdam UMC Biobank Core Facility, Amsterdam UMC, Amsterdam, The Netherlands; 8Department of Radiology, Amsterdam UMC, Amsterdam, The Netherlands; 9Department of Medical Microbiology, Amsterdam UMC, Amsterdam, The Netherlands; 10Department of Internal Medicine, Amsterdam UMC, Amsterdam, The Netherlands; 11Neurochemical Laboratory, Amsterdam UMC, Amsterdam, The Netherlands; 12Department of Clinical Chemistry, Amsterdam UMC, Amsterdam, The Netherlands;
13Department of Clinical Epidemiology, Biostatistics and Bioinformatics, Amsterdam UMC, Amsterdam, The Netherlands.
