# Supplementary material for: Neurofilament light increases over time in severe COVID-19 and is associated with delirium
Source: Brain Commun. 2022 Jul 26;4(4):fcac195. doi: 10.1093/braincomms/fcac195 (PMC9351727; doi:10.1093/braincomms/fcac195)
Supplement: fcac195_Supplementary_Data [file fcac195_supplementary_data.zip › Supplementary_material_statistical_models.docx]

**Statistical models from:**

*Smeele, Vermunt et. Al, Neurofilament light increases over time in severe COVID-19 and is associated with delirium.*

library(readxl), library(readr) library(tidyverse) library(lme4) library(lmerTest) library(ggplot2) library(gridExtra) library(ggpubr) library(emmeans) library(splines) library(ggeffects) library(rstatix)

*#1. Model for NfL over time and extraction of the time point when the slopes stop increasing significantly and at which time point the groups differ from each other in slope and/or NfL levels:*

model_1_NfL_over_time <-

lmer(log(NfL) ~ bs(days_since_icu_admission, df=3) +

age + log(creatinine) +

(1|patient_id) + (0+days_since_icu_admission | patient_id),

data = longitudinal_data_NfL)

model_2_NfL_over_time_mortality_group <-

lmer(log(NfL_levels) ~ bs(days_since_icu_admission, df=3)*survival_group+

age + log(creatinine) +

(1|patient_id) + (0+days_since_icu_admission | patient_id),

data = longitudinal_data_NfL)

emmeans(model_2_NfL_over_time_mortality_group,

pairwise ~ mortality_group | days_since_icu_admission,

at = list(days_since_icu_admission = 0:27), params = "deg", type='re')

emtrends(model_2_NfL_over_time_mortality_group,

pairwise ~ mortality_group | days_since_icu_admission ,

var = "days_since_icu_admission",

at = list(days_since_icu_admission = 0:27), params = "deg", type='re')

*#2. Models predicting clinical outcomes (mortality or delirium) with NfL at admission or peak NfL, and next testing the correlations after stratification by age.*

glm(clinical_outcome ~ log(NfL) +

age + log(creatinine),

data = NfL_data_admission_or_peak_timepoint, family= 'binomial'))

cor_test(clinical_outcome, NfL, method=c("spearman"),

data=data_subset_age)

# 3. *Individuals were split into tertile groups based on the values of the following markers: sequential organ failure assessment score, IL-1β, IL-6, IL-8, TNF-α, d-dimer. The tertile groups were then included as a fixed effect and for its interaction with time after baseline in linear mixed models for the NfL trajectory over time.*

model_NfL_over_time_by_tertile_marker <-

lmer(log(NfL) ~ bs(days_since_icu_admission,df=3)*SOFA_inflammation_ddimer_tertile_group+

age+ log(creatinine)+

(1|patient_id) + (0+days_since_icu_admission | patient_id),

data = longitudinal_data_NfL)

# to make the graphs:

prediction_NfL_with_tertiles_other_markers <-

ggpredict(model_NfL_over_time_by_tertile_marker,

terms = c("days_since_icu_admission [0:27]",

"SOFA_inflammation_ddimer_tertile_group "), type = "re")

# and to test if the NfL estimates and/or slopes at any time point differ significantly from each other.

emmeans(model_NfL_over_time_by_tertile_marker,

pairwise ~ SOFA_inflammation_ddimer_tertile_group|days_since_icu_admission,

at = list(days_since_icu_admission = c(0:27)), params = "deg", type='re')

emtrends(model_NfL_over_time_by_tertile_marker,

pairwise ~ SOFA_inflammation_ddimer_tertile_group | days_since_icu_admission, var = "days_since_icu_admission",

at = list(days_since_icu_admission = 0:27), params = "deg", type='re')

**MGH cohort, data available publically** - [MGH Covid Study Overview (olink.com)](https://info.olink.com/mgh-covid-study-overview-page?hsCtaTracking=fff99a2a-81c1-4e4a-a70d-6922d26503b4%7C202c2809-0976-48f7-aad0-3903c36624ca)

library(lme4), library(lmerTest), library(tidyverse), library(ggpubr), library(gridExtra), library(emmeans), library(splines), library(ggeffects), library(tableone)

*# 1. NfL over time (corrected for age + creatinine) with group effects*

model_NfL_mgh_long <-

lmer(NfL_npx ~ days_after_admission*clinical_groups+

age.cat+ creatinine +

(1+days_after_admission|subject_id),

data=mgh_data_NfL)

*# Difference at baseline and slope between groups explored using the following.*

emmeans(model_NfL_mgh_long,

pairwise ~ clinical_groups:days_after_admisison,

at = list(days_after_admission=0), var = "days_after_admission")

emtrends(model_NfL_mgh_long,

pairwise ~ clinical_groups, var = "days_after_admission")

*# 2. Baseline NfL as independent predictor for mortality, after adjustment for age + creatinine*

glm(mortality_group ~ NfL_npx+

age.cat+ creatinine, family='binomial' ,

data = mgh_data_NfL)

*# 3. NfL and markers for inflammation/coagulation. Groups made based on tertile values.*

inflammation_or_coagulation_marker__model <-

lmer(NfL_npx ~ inflammation_orcoagulation_marker*days_after_admission+

age.cat+ creatinine+

(1|subject_id)+ (0+days_after_admission|subject_id),

data = mgh_data_NfL)

*# Differences per time point explored using the following:*

emmeans(inflammation_or_coagulation_marker_model,

pairwise ~ inflammation_or_coagulation_marker | days_after_admission,

at = list(days_after_admission=0), var = "days_after_admission")

emtrends(inflammation/coagulation_marker_model,

pairwise ~ inflammation_or_coagulation_marker, var = "days_after_admission")

Caption : Caption : Figure 1: Flow chart showing number of included patients and number of samples. MGH = Massachusetts general hospital
