# Supplementary material for: Neurofilament light increases over time in severe COVID-19 and is associated with delirium
Source: Brain Commun. 2022 Jul 26;4(4):fcac195. doi: 10.1093/braincomms/fcac195 (PMC9351727; doi:10.1093/braincomms/fcac195)
Supplement: fcac195_Supplementary_Data [file fcac195_supplementary_data.zip › Supplementary figures and legends_Smeele et al_NfL in COVID_12-07-2022.pdf]

Smeele, Vermunt et. Al, Neurofilament light increases over time in severe COVID-19 and is associated with delirium.

Supplementary Figure 1: Baseline correlations spearman

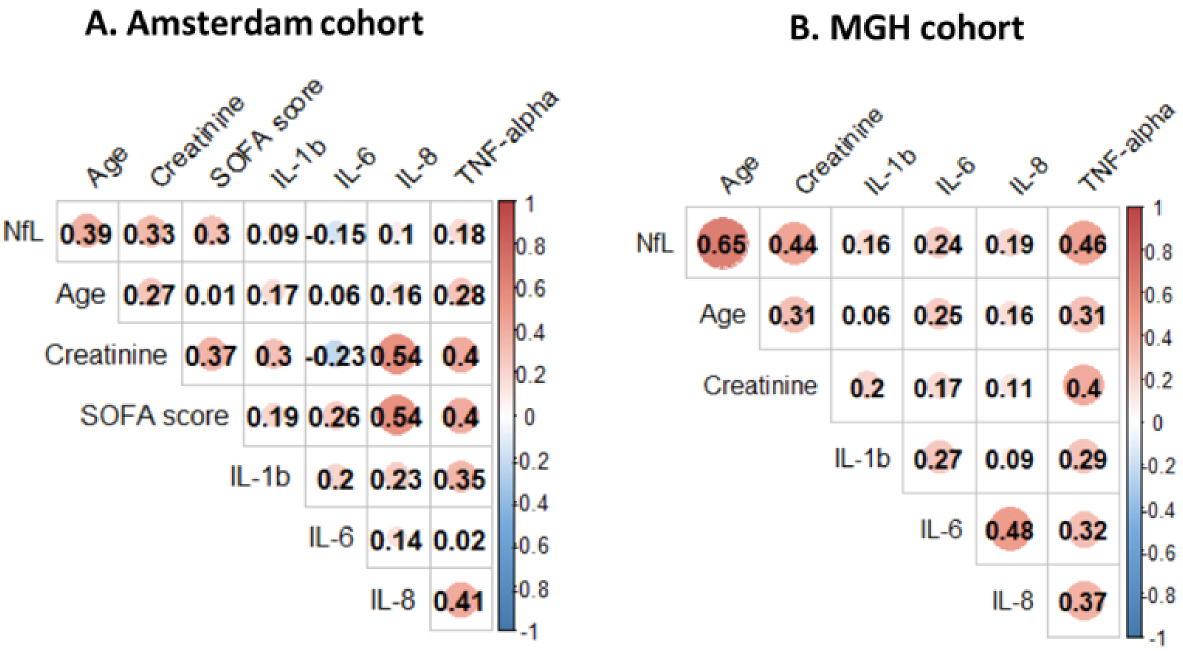

Supplementary Figure 1: Spearman correlation between baseline markers. Association between several markers, including NfL, Age, Creatinine, sequential organ assessment score (SOFA), IL-1β, IL-6, IL_8, and TNF-α (A) Amsterdam cohort and (**B**) MGH cohort. Values shown represent the rho of each correlation, red color implies significant correlation with p < 0.05.

Supplementary Figure 2: Neurofilament light over time in COVID-19. Raw data

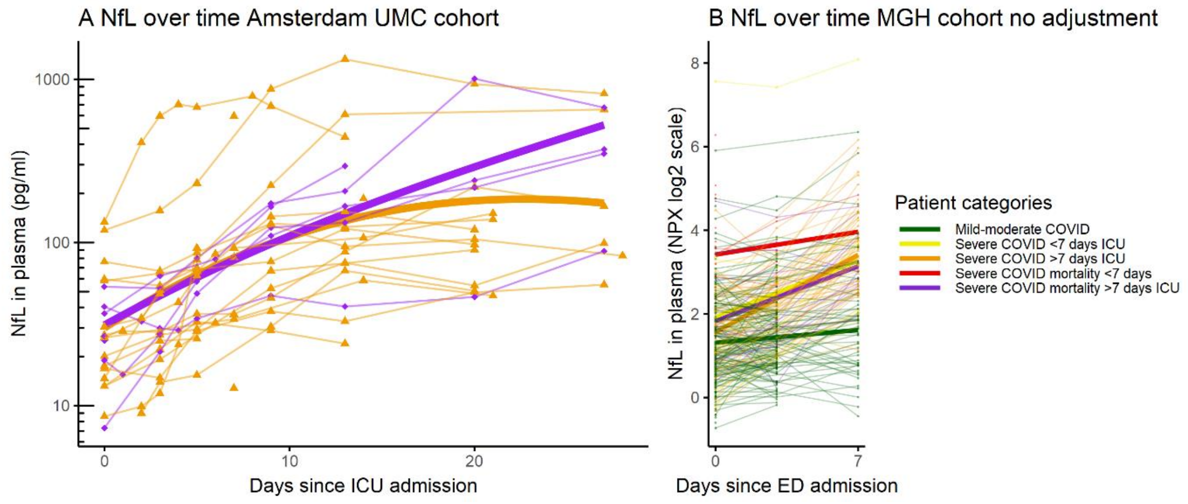

Supplementary Figure 2: Neurofilament light (NfL) over time in COVID-19. Each line represents the individual trajectory of a patient. (**A**) Intensive care unit (ICU) patients with >7 days of admission, admitted to Amsterdam University Medical Centre. Orange: survival at 90 days, Purple: deceased at 90 days. Linear mixed model, marginal means show slopes significantly differ from day 17 until day 25, t-ratio= -2.17, p= 0.04 and t-ratio=-2.06 p= 0.04 respectively. (**B**) Patients admitted to the Emergency Department (ED) of Massachusets General Hospital (MGH). Analysed using lineair mixed models: Green: Mild-moderate COVID-19 (used as reference group, t-value = 4.422, p < 0.001), Yellow: Severe COVID-19 with ICU admission and discharge before day 7 (t= 2.53 p= 0.01). Orange: Severe COVID-19 with ICU admission and discharge after day 7 (t= 8.32, p<0.001). Red: Severe COVID-19 with ICU admission and mortality before day 7 (t= 0.54, p= 0.59), Purple: Severe COVID-19 with ICU admission and mortality after day 7 (t= 3,23, p= 0.001).

Supplementary Figure 3: Inflammation markers

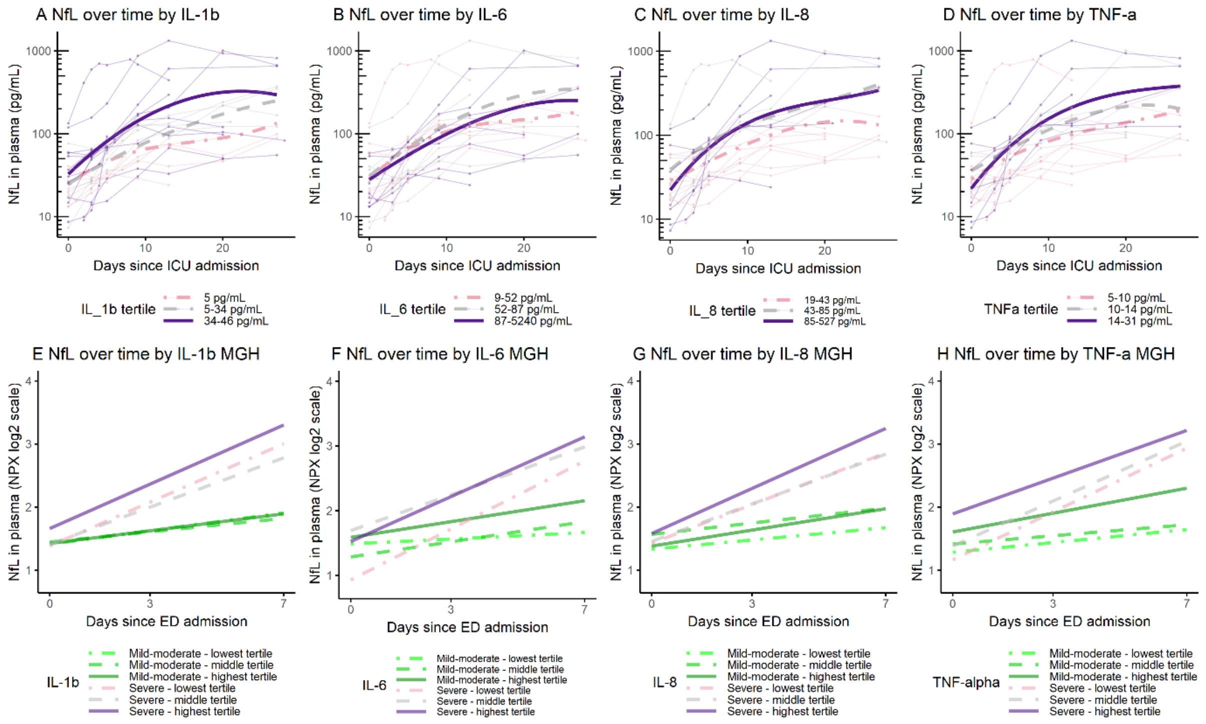

Supplementary Figure 3: Association of baseline inflammation markers on the trajectory of NfL. Analysed using linear mixed models to estimate the NfL slopes over time for severe COVID-19 tertile groups based on inflammation markers at admission A-D: AmsterdamUMC cohort, showing (A)IL-1β: Slopes differed between the upper and lower tertile between day 6 and day 9 (t-ratio= -2.59, p= 0.04 and t-ratio= -2.46, p= 0.05 respectively). Slopes stopped increasing significantly for the lowest tertile on day 10, the middle tertile on day 23, and the highest tertile on day 19. (B) IL-6: Slopes did not differ significantly between IL6 tertile groups. The slopes stopped increasing significantly for the lowest tertile on day 13, for the middle and upper tertile on day 21. (C)IL-8: Slopes differed significantly for the lowest and highest tertile from day 0 to day 6 (t-ratio= -2.78, p= 0.02 and t-ratio= -2.43, p= 0.05 respectively). Slopes stopped increasing significantly on day 19 for the lowest tertile, day 23 for the middle tertile and day 18 for the highest tertile (D) TNF-α: Slopes differ significantly between the highest and middle tertile from day 0 until day 6 (t-ratio= -2.38, p= 0.05, t-ratio=

-2.42, p= 0.05, respectively), between the highest and lowest tertile between day 2 and day 8 (t-ratio= -2.64, p= 0.02 and t-ratio= -2.66, p= 0.03 respectively). Slopes stopped increasing on day 22 for the lowest tertile, day 19 for the middle tertile, and day 20 for the highest tertile.E-H: Massachusetts general hospital cohort, showing IL-1b, IL-6, IL-8, TNF-α respectively. Analysed using linear mixed models and marginal means to estimate the NfL slopes over time for both mild-moderate and severe COVID-19 tertile groups based on inflammation markers at admission. For IL-1β, IL-6 and IL-8 the severe COVID-19 tertile groups had significantly steeper slopes than the mild-moderate COVID-19 tertiles groups (E) IL-1β: t-ratio between -6.25 and -3.21, all p-values <0.01. (F) IL-6: t-ratio between -5.78 and -3.30, all p-values <0.01. (G) IL-8: t-ratio between -6.13 and -2.92, all p-values <0.01. For (H) TNF-α this was similarly the case (t-ratio between -6.30 and -2.91, all p-values <0.01) except that the slope of the highest mild-moderate COVID-19 tertile did not differ from the highest severe COVID-19 tertile (t-ratio -2.57, p= 0.108). IL = Interleukin, TNF = Tumor Necrosis factor
