# Supplementary figures and images for: Neurofilament light increases over time in severe COVID-19 and is associated with delirium
Source: Brain Commun. 2022 Jul 26;4(4):fcac195. doi: 10.1093/braincomms/fcac195 (PMC9351727; doi:10.1093/braincomms/fcac195)

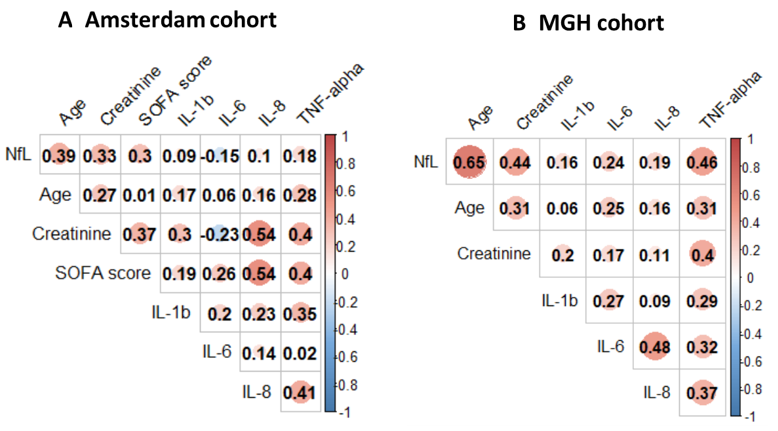

Supplement: fcac195_Supplementary_Data [file fcac195_supplementary_data.zip › Supplementary_Figure 1.png]

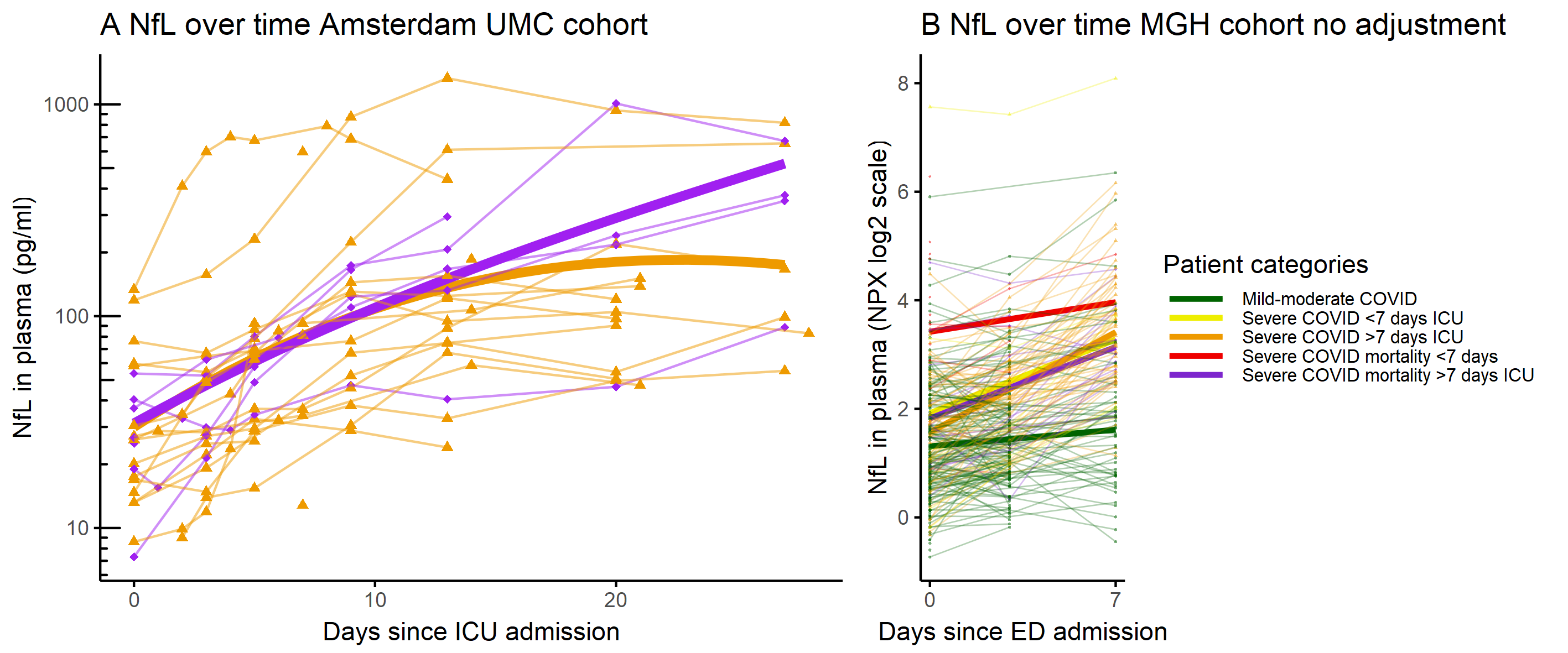

Supplement: fcac195_Supplementary_Data [file fcac195_supplementary_data.zip › Supplementary_Figure 2.png]

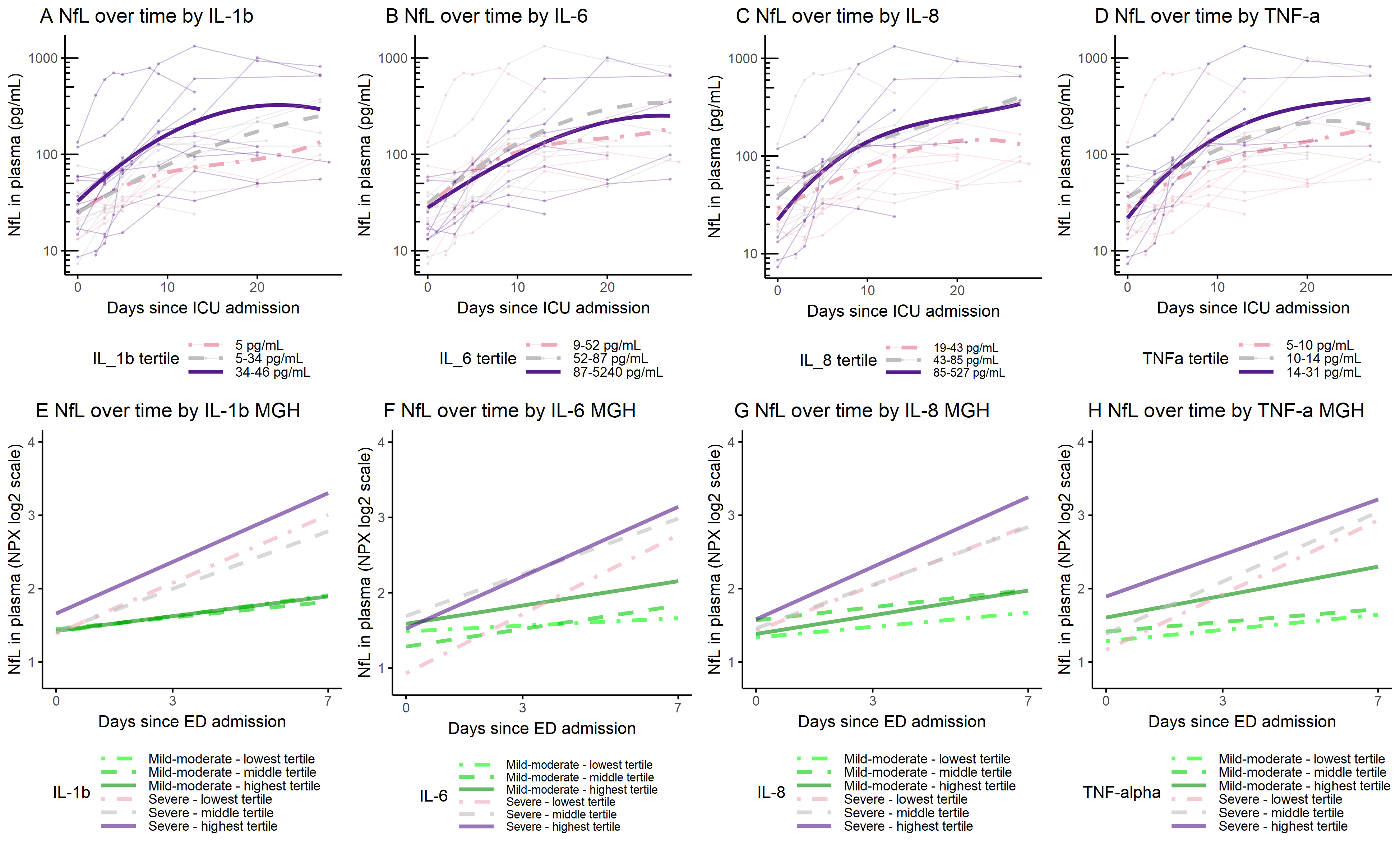

Supplement: fcac195_Supplementary_Data [file fcac195_supplementary_data.zip › Supplementary_Figure 3.png]
